# Data‐Driven Polyoxometalate Chemistry

**DOI:** 10.1002/chem.202501528

**Published:** 2025-09-07

**Authors:** Aleksandar Kondinski, Nadiia I. Gumerova, Annette Rompel, Paolo Falcaro, Tobias Schreck

**Affiliations:** ^1^ Institute of Physical and Theoretical Chemistry Graz University of Technology Stremayrgasse 9 Graz 8010 Austria; ^2^ Fakultät für Chemie, Institut für Biophysikalische Chemie Universität Wien Josef‐Holaubek‐Platz 2 Vienna 1090 Austria; ^3^ Institute of Visual Computing Graz University of Technology Inffeldgasse 16c Graz 8010 Austria

**Keywords:** artificial intelligence, cheminformatics, digital chemistry, fair data, polyoxometalate

## Abstract

Polyoxometalates (POMs) are nanoscale, structurally versatile metal–oxo clusters with emerging applications in sustainability, energy, nanoelectronics, and life science technologies. Owing to their structural complexity, some all‐inorganic POMs are often perceived as serendipitous outcomes from self‐assembly processes, which poses challenges for scalable rational design. From this perspective, we therefore examine how the development of POM informatics and, more generally, data‐driven POM exploration can pave the way for the molecular engineering of new POM‐based materials targeting customized applications. In the process, we highlight recent successes in the digitalization of POM chemistry and outline which advanced technologies are necessary for progress in this promising area of research.

## Graphical abstract

An integrative data‐driven approach leveraging AI, computations, and robotic experimentation enables accelerated discovery of polyoxometalates, bridging synthetic unpredictability and paving the way for rational, predictive, and functional cluster chemistry.

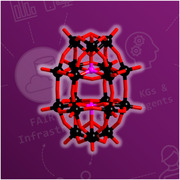

## 1 Introduction

Polyoxometalates (POMs) are discrete, highly charged nanomolecular metal‐oxo clusters, typically built of early transition metals in high oxidation states.^[^ ^1^ ^]^ POMs exhibit a wide range of structural and compositional versatility, ranging from small tri‐ or tetrametalate clusters to giant assemblies composed of dozens or even hundreds of metal centers.^[^ ^2^ ^]^ Their modular composition and tunable oxidation states make them suitable for application in catalysis, molecular electronics, energy storage, and life science.^[^ ^3^, ^4^, ^5^ ^]^ Moreover, POMs serve as integral building blocks for extended solids and hybrid materials, where they retain their redox‐active and structural functionalities.^[^ ^6^ ^]^

**Figure 1:**
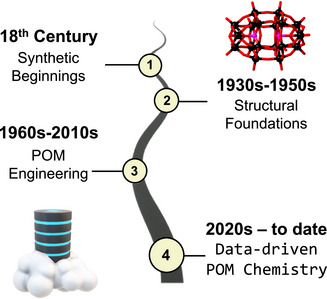
Compact timeline of the evolution of POM chemistry.

POM chemistry has been gradually evolving for over two centuries (see Figure 1). Pioneering chemists such as Scheele and Berzelius had already noted the formation and synthesis of POMs in the late 18^th^ and early 19^th^ centuries; however, their structuring remained unknown until the early 20^th^ century.^[^ ^7^, ^8^ ^]^ The foundations of coordination chemistry by Alfred Werner have rekindled interest in this aspect of POMs, motivating various structural proposals and ultimately the first crystallographic elucidations.^[^ ^8^, ^9^ ^]^ In this regard, many common “classical” POM archetypical structures such as Keggin, Lindqvist, Wells–Dawson, octamolybdate, paratungstate, and decavanadate, were first reported during the 1930s and 1950s, which marks a second stage in the development of POM chemistry.^[^ ^8^, ^9^ ^]^ Fundamental interest in POMs, as well as applications in catalysis have been driving the molecular engineering of POMs since the 1950s onward.^[^ ^10^ ^]^ In this third period of developments, the number of known POMs has significantly expanded. Also, from the early 2000s, advances in computational chemistry and computer technologies have created opportunities for a more comprehensive understanding of the intrinsic stability of POMs, their electronic and spectroscopic properties in relation to experimental observations.^[^ ^11^ ^]^ With the rapid digitization over the past few years and global access to advanced artificial intelligence (AI) tools, POM chemistry and POM‐based technologies are gradually entering a new fourth “digital” or “data‐driven” stage, to which, inspired by the parallels of the Fourth Industrial Revolution, we may even refer to as “POM 4.0.”^[^ ^12^ ^]^

In this perspective, we, therefore, examine the immediate challenges for data‐driven POM chemistry, starting with the necessity to express POM information in a machine‐actionable format. This necessity requires the urgent development of polyoxometalate cheminformatics, that is, the computational and informational technique toolset that will enable the solving of polyoxometalate chemical problems. Polyoxometalate cheminformatics will naturally also encompass standardizations capturing the caveats of POM structure representation, the complexity of POM‐based processes and the dynamics of POM interconversions. Although applications of artificial intelligence (AI) have enabled advances in POM chemistry in recent years, wider standardization and semantic structure description enable laying the foundations for automated, evidence‐based reasoning as part of broad knowledge systems.^[^ ^13^ ^]^ Formalization of the POM structural and electronic features is also a step for understanding the self‐organization and reactivity of the building block constituents (e.g. multimetal–oxo fragments) that may be similar across different structures or show some correlated activity behavior (e.g., catalaphores, toxicophores, etc.) Finally, accelerating the frontier of data‐driven POM chemistry comes with the adaptation and tailoring of AI technologies, aspects that are addressed in this work.

## 2 Polyoxometalate Cheminformatics

**Figure 2:**
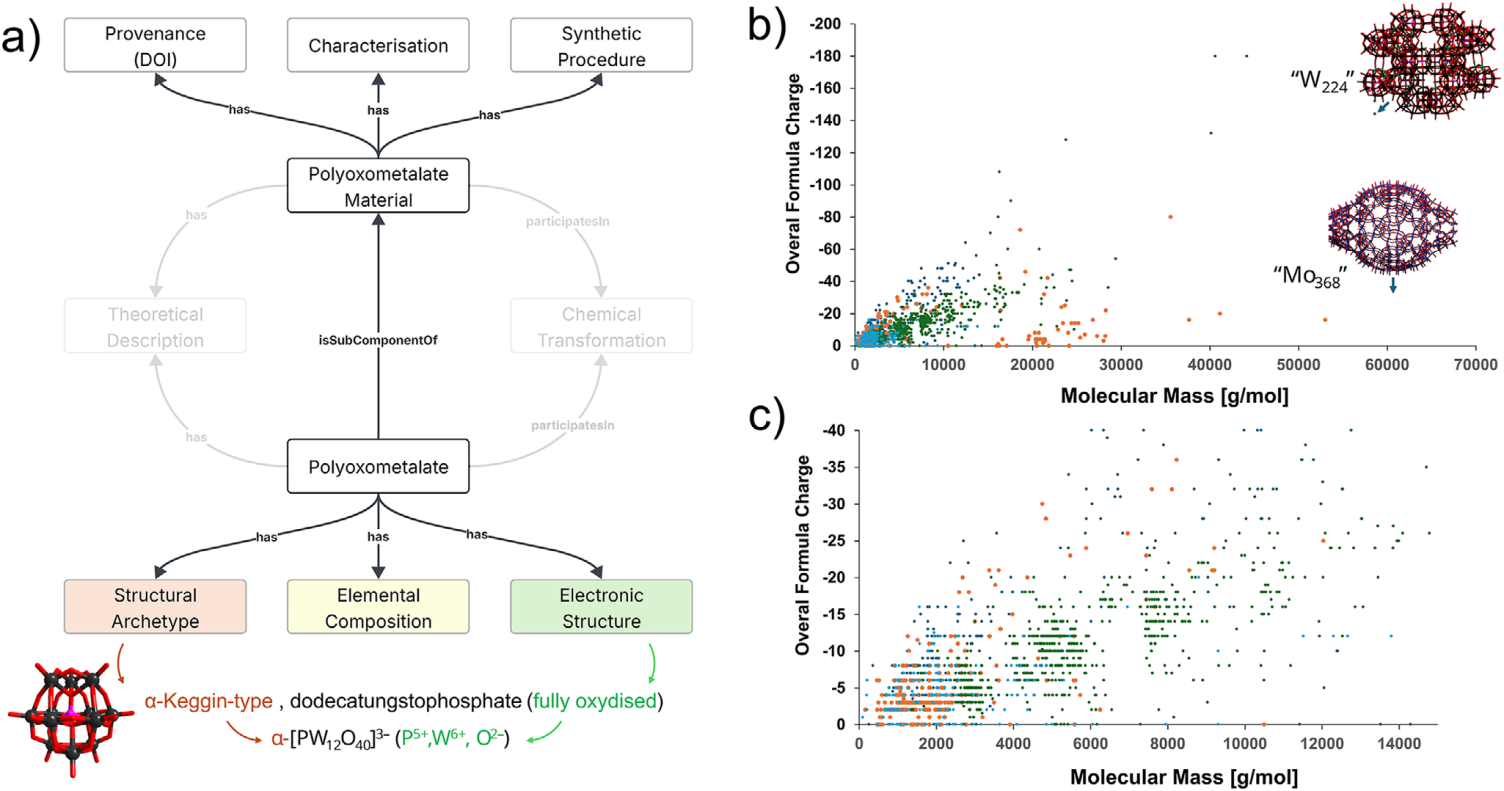
a) Schematic representation of the herein proposed three key concepts guiding further taxonification of POMs. Note that POMs are entities or subcomponent of typically crystaline POM Materials. Both POMs and POM materials can a subject of theoretical investigations or considered as components of chemical transformations. b) A scatter plot from a curated dataset^[^ ^20^ ^]^ of POM instances, comparing overall formula charge and molecular mass. c) A “zoomed in” version of plot under point (b), with molecular masses up to 30,000 Da. Points are color‐coded by the main addenda metal (e.g., W = green, Mo = orange, V = light blue, and other metals shown in dark blue). Examples of some representative high‐nuclearity structures are shown in b).

Information denotes structured data that document the properties of entities, whereas knowledge comprises the logical relations among those data that explain causality.^[^ ^13^ ^]^ Robust decision‐making in chemistry and the symbolic‐AI systems that support it therefore require rigorously defined formal representations of chemical concepts and instances, a need that historically drove the emergence of cheminformatics.^[^ ^13^ ^]^ POMs as inorganic entities featuring complex coordination and bonding, remain challenging to address *via* common cheminformatics identifiers.^[^ ^14^ ^]^ However, much of their subfamily taxonomization goes along three main factors (see Figure 2a): i) elemental composition, ii) electronic structure, and iii) generic archetype topology. Systematic explorations of POM reactivity require fine‐tuning of a single factor across a series of POM instances. However, this often may be challenging, as the three factors are not fully independent from one another and often influence each other. POMs can be classified according to their elemental composition, specifically by the dominant addenda element (e.g., polyoxotungstates, polyoxomolybdates, polyoxovanadates, etc.). The compositional dimension also provides distinction between an “iso‐” or “hetero‐”, “mixed‐metal” (i.e., “mixed‐addenda”), “oxo‐substituted”, protonated, “hybrid”, or other type of POM instances.^[^ ^15^, ^16^ ^]^ In addition to the elemental aspect, the structural POM archetype refers to a canonical arrangement of the metal–oxo building blocks, which can be seen as analogous to the concept of “frameworks” in the context of zeolite and reticular chemistry.^[^ ^17^ ^]^ Well‐known examples include the Keggin 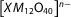 and Wells–Dawson 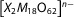 archetypes. Some archetypes can be encountered for different addenda element‐based POM, which often allows the construction of mixed metal systems with tailored properties.^[^ ^9^ ^]^ On the other hand, there are also archetypes that appear idiosyncratic and often strictly linked to a particular addenda element or a molecular composition. In more structurally complex POM, different building block combinations may favor the formation of distinct archetypes, depending on reactivity and availability.^[^ ^18^ ^]^ The local coordination, which also stipulates the number of terminal oxo/hydroxo/aqua ligands *per* addenda center, is a reflection of the particular archetype, and it directly affects the structural integrity of the POM to be involved in (multielectron) redox processes. Finally, the electronic structure of the POM, which includes the total overall charge and its distribution, is another important aspect, as it provides differentiation between “fully oxidized”, “partially reduced,” and fully, highly and super‐reduced POMs.^[^ ^19^ ^]^ The charge configuration and its (de)localization can further affect the magnetic and internal bonding properties, as metal–metal bonding can, for some (partially) reduced structures.^[^ ^4^ ^]^

As mentioned earlier, standard chemical identifiers for organic molecules, such as the Simplified Molecular‐Input Line‐Entry System (SMILES) or the International Chemical Identifier (InChI), often struggle with complex inorganic species such as POMs.^[^ ^14^ ^]^ Features like bridging oxo ligands, protonation equilibria, mixed metal oxidation states, and open‐shell electrons are generally difficult to encode in standard string‐based representations. The placement of protons on bridging versus terminal oxygens can alter the observed structure considerably, yet conventional encodings lack a robust syntax for capturing these subtle differences. Similarly, partially reduced POMs may exhibit electron delocalization, variable metal–oxo bond orders, or open‐shell spin states. One strategy to address these challenges is to develop extended encodings specifically designed for inorganic systems or to implement hybrid approaches that integrate both geometry and electronic structure. In this regard, the classical *Chemical Markup Language* (CML) which is based on an eXtensible Markup Language (XML) format that can include specialized tags for redox states, bridging ligands, and other elaborate features, may provide a suitable and rather flexible information framework for describing POMs.^[^ ^21^ ^]^ However, its verbosity, steeper learning curve, and the need for carefully maintained domain‐specific schemas can limit broader adoption.

The recently curated dataset of POM formulas covering the representation of nearly 2000 POM formulas (see Figure 2b,c), is a step toward the development of POM cheminformatics.^[^ ^20^ ^]^ For each POM formula instance, the dataset also incorporates linked information such as literature provenance, total charge, molecular mass, and list of constituting elements, allowing programmatic search and comparisons. Plots of charge versus molecular mass can be indicative of stability limits in super‐ and undercharged POMs. Future augmentation of this curated POM dataset with electronic information, such as oxidation states of different elements, synthetic information, and reactivity, can be of high interest not only for the development of a comprehensive POM knowledge base, but also for predictive analytics. Among various considerations, the identification and formal representation of POM archetypes is crucial not only for clustering POM instances,^[^ ^22^ ^]^ but also for applying rational approaches to understand and discover new ones.

## 3 Current Trends—AI for POMs

Despite the need for comprehensive POM cheminformatics, AI techniques have been more frequently leveraged at different stages of POM discovery over the past few years. One prominent example is the use of artificial neural networks (ANNs) for modelng POM solubility. Rahman et al.^[^ ^23^ ^]^ developed an ANN incorporating a physically motivated σ‐profile descriptor to capture how alkali cations modulate aqueous solubility across diverse POM salts, revealing three main trends—“normal,” “anomalous,” and an experimentally validated “amphoteric” regime exemplified by [${\mathrm{Ti}}_{6}$ 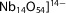. Complementing such predictive frameworks, Skjærvø et al.^[^ ^24^ ^]^ introduced an explainable machine learning (ML) approach, *ML‐MotEx*, to interpret time‐resolved crystallization pathways from Pair Distribution Function (PDF) data of transition‐metal tungstates, systematically fitting thousands of candidate POM‐like fragments and applying SHapley Additive exPlanation (SHAP) to identify the most critical Keggin‐type building blocks. Their analysis linked more‐ordered POM precursors (e.g. Fe–W compositions) to rapid crystallization, whereas disordered Co/Ni analogs stalled as amorphous intermediates, thereby highlighting mechanistic connections between local POM motifs and global kinetics. Meanwhile, Anker et al.^[^ ^25^ ^]^ introduced *POMFinder*, a tree‐based eXtreme Gradient Boosting (XGBoost) classifier trained on an extensive simulated POM database, demonstrating 94% accuracy in classifying experimentally measured PDFs, including fast‐acquisition or in situ data, with SHAP analysis pinpointing metal–metal distances as dominant predictive features. Beyond solubility and crystallization, Dan et al.^[^ ^26^ ^]^ showed that unsupervised pipelines can extract hierarchical motifs directly from high‐resolution electron microscopy images using a “classify‐then‐compose” scheme based on rotationally invariant Zernike descriptors. Their algorithm uncovered a novel pentagonal arrangement of metal–oxide octahedra in a Mo–V–Te–Nb mixed‐metal oxide, showcasing how motif‐level insights help identify hidden structural phases and possible self‐assembly routes.

On the other hand, modular robotic platforms have obtained increasing interest in the context of automated discovery and synthesis of POM materials by integrating reconfigurable hardware with sophisticated algorithmic control.^[^ ^27^, ^28^ ^]^ These systems employ standardized interfaces and programmable reaction modules that execute precise fluid handling, real‐time monitoring, and adaptive condition adjustments based on feedback loops.^[^ ^28^ ^]^ Algorithm‐driven robots enable high‐throughput experimentation across complex synthesis spaces. For instance, an autonomous platform adopted probabilistic search methods with the aim of exoloring electrochemical routes for inorganic species (including POM clusters) with minimal human intervention.^[^ ^29^ ^]^ Reinforcement learning and active learning strategies have been used to refine reaction parameters in closed‐loop workflows, leading to the discovery of novel POM–MOF structures through iterative screening guided by uncertainty metrics.^[^ ^27^ ^]^ Human‐in‐the‐loop approaches can further enhance efficiency. Although fully autonomous systems accelerate the identification of “gigantic” POM clusters, collaborative algorithms that combine human intuition with machine learning have outperformed strategies relying solely on either robots or humans.^[^ ^30^, ^31^ ^]^ By using adaptive AI models, such as Bayesian optimization and neural networks, robots can map vast experimental conditions swiftly, refining hypotheses on‐the‐fly to arrive at optimal or unexpected targets. This synergy between modular robotic architectures and state‐of‐the‐art machine learning frameworks offers a transformative pathway for accelerating POM discovery, ensuring more reproducible, data‐rich experimentation, and enabling breakthroughs in inorganic materials research that would be exceedingly difficult through manual methods alone.

## 4 Benefits—AI for “POM‐Tech”

The nanoscopic size and redox‐responsive properties of POMs, make them promising candidates across multiple technological frontiers.^[^ ^27^, ^30^, ^32^ ^]^ In catalytic applications, POMs excel at facilitating (photo)oxidations and multi‐electron processes, partly due to their strong Brønsted acidity and redox tunability.^[^ ^32^ ^]^ However, the coexistence of multiple reactive sites and oxidation states complicates the mechanistic elucidation, necessitating data‐driven approaches to correlate structural motifs with catalytic performance.^[^ ^28^, ^33^ ^]^ Neural‐network models can predict key descriptors (e.g., deprotonation enthalpies, electron affinity) and thereby pinpoint “catalophores” that promote higher turnover frequencies or improved stability under reaction conditions.^[^ ^34^ ^]^ Recent breakthroughs in interpretable machine learning for oxide‐based catalysis have enabled the identification of structural “genes,” providing transparent design rules for active sites.^[^ ^33^ ^]^

Another application area is “POMtronics,” where POM clusters are deployed in molecular electronics and neuromorphic architectures.^[^ ^35^ ^]^ Because these clusters can be reversibly oxidized or reduced in molecular junctions, they afford large and stable modulations of conductance for memory and switching functionalities.^[^ ^35^ ^]^ Advanced machine‐learning techniques (e.g., unsupervised clustering) have proven adept at analyzing the extensive current–voltage datasets generated, revealing subtle structure–property correlations that guide device optimization.^[^ ^35^ ^]^ Furthermore, POM‐assisted ionic migration can enhance memristor performance, a crucial step toward realizing reservoir computing systems.^[^ ^36^ ^]^ Drawing on established pipelines from organic electronics,^[^ ^34^ ^]^ one can explore redox‐active motifs and next‐generation (hybrid) POM‐based electronic devices.

Finally, POMs are emerging as essential components of extended solids and biocomposites, leveraging their robust frameworks and potential bioactivity.^[^ ^5^, ^37^, ^38^, ^39^, ^40^ ^]^ Their integration into proteins, polymers, or nanoparticles can yield antibacterial, antiviral, or anticancer effects, as well as enzyme‐like activity.^[^ ^41^, ^42^, ^43^ ^]^ Notably, the formation of POM‐protein assemblies has facilitated protein crystallography, while the regioselective oxidative cleavage illustrates the artificial‐metalloenzyme functionality of certain POM clusters.^[^ ^44^ ^]^ Rational design of POM‐biocomposites remains challenging, as even minor alterations in cluster composition and reaction conditions can often produce major shifts in biological reactivity.^[^ ^40^ ^]^ Moreover, the solution behavior of POMs, including partial hydrolysis, decomposition products, and the presence of heavy metals such as tungsten and vanadium, requires thorough toxicity assessment.^[^ ^45^ ^]^ Data‐driven methods such as biomedical knowledge graphs can clarify structure–activity relationships and streamline toxicity predictions, as demonstrated by advances in drug discovery.^[^ ^46^ ^]^

Although machine learning has already produced valuable insights, most current models are trained on narrow and sometimes imbalanced data sets, and this limits both accuracy and transferability. Studies on porous framework materials show that performance plateaus at modest data volumes and motivate transfer learning and data augmentation.^[^ ^47^ ^]^ Hybrid workflows that combine data driven learners with domain knowledge captured in large‐scale knowledge graphs widen chemical coverage and improve confidence estimates.^[^ ^48^ ^]^ Surveys of language models for chemistry recommend attaching explicit uncertainty metrics when data are sparse.^[^ ^49^ ^]^ Together with progress in knowledge engineering, these developments indicate that combining symbolic reasoning with machine learning can deliver more reliable predictions for POM chemistry in the near future.^[^ ^13^ ^]^

## 5 Critical Enablers for POM 4.0

### 5.1 Knowledge Graphs and Large Language Models

The combination of large language models (LLMs) and knowledge graphs (KGs) has significantly advanced reticular chemistry, especially in the design and discovery of metal–organic frameworks (MOFs). For example, Zheng et al. introduced a “GPT‐4 Reticular Chemist” framework that operates through a cooperative AI–human workflow for synthesis planning and characterization, thus successfully guiding the discovery of structurally isoreticular series of MOFs.^[^ ^50^ ^]^ Similarly, An et al. developed the MOF Knowledge Graph (MOF‐KG) alongside a benchmark dataset for Knowledge Graph Question Answering in Materials Science (KGQA4MAT), that enables domain experts to query complex chemical data in a natural language format.^[^ ^51^ ^]^ These studies showcase the synergy between LLMs and KGs. LLMs are well‐suited for interpreting unstructured text and generating hypotheses, while KGs provide a structured and consistent framework that supports data accessibility and precise querying. In addition, LLMs can assist in formulating complex queries, particularly when users are not fully familiar with the structure of the knowledge graph.

**Figure 3:**
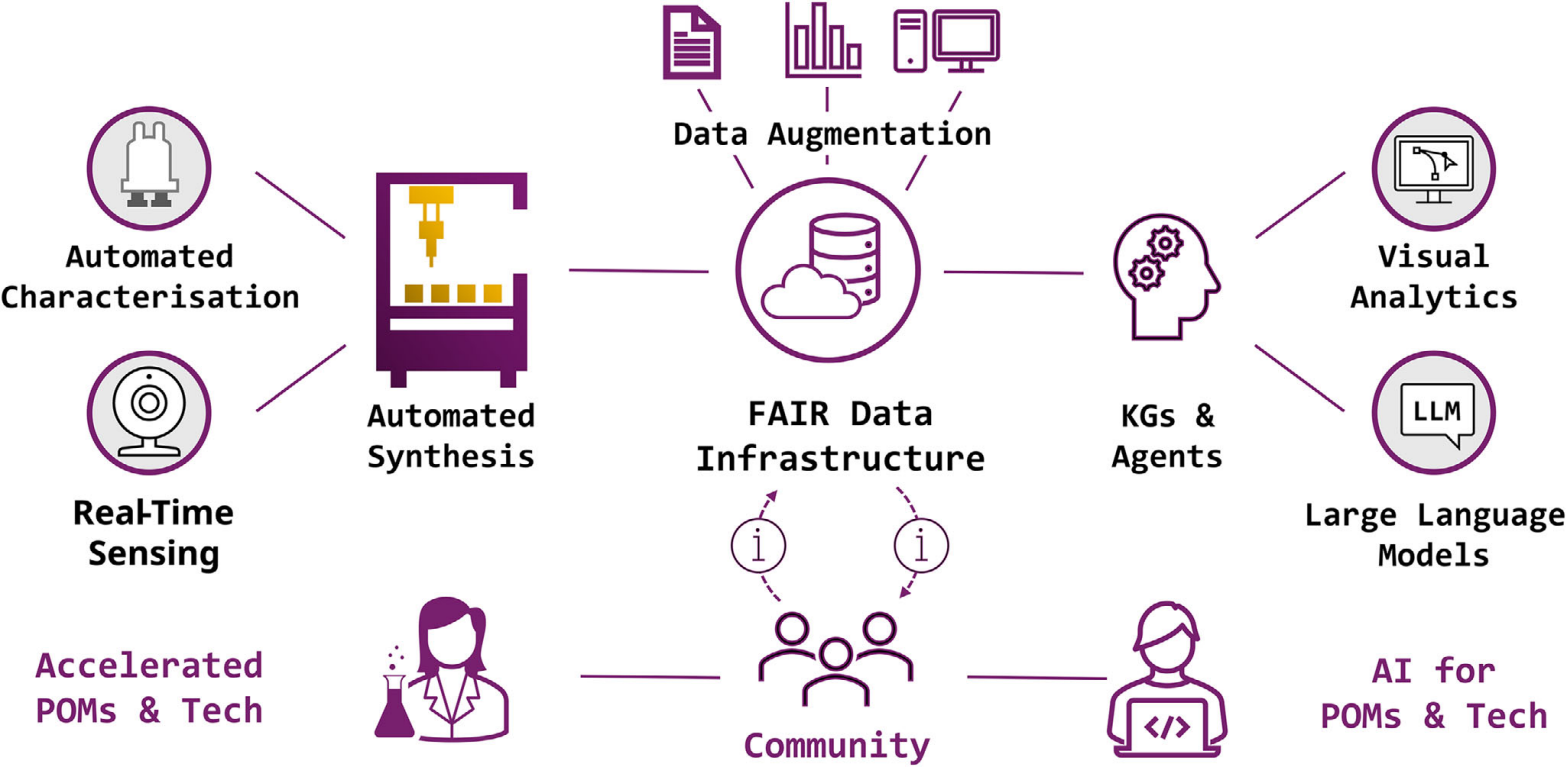
Schematic overview of an envisioned data‐driven ecosystem for POM 4.0, with a findable, accessible, interoperable, and reusable (FAIR) data infrastructure at its core. Automated synthesis and real‐time characterization feed experimental data into centralized repositories, enabling data augmentation and knowledge graph (KG)–driven analyses. Large Language Models (LLMs) and AI agents interpret this information, guided by visual analytics tools that engage researchers in the loop.

Beyond reticular chemistry, similar integrations have advanced progress in a broad range of chemical fields. ChemCrow, which is an LLM‐based chemistry agent, demonstrates autonomous performance in organic synthesis, drug discovery, and materials design by coupling natural language generation with specialized toolkits.^[^ ^52^ ^]^ KG approaches have also proven effective for metal–organic polyhedra and zeolitic frameworks, demonstrating their applicability.^[^ ^53^, ^54^ ^]^ For POM research, domain‐specific KGs that capture detailed experimental and computational information can serve as a robust foundation for AI‐driven workflows, while LLMs can interpret existing literature, guide new experiments, and help verify results. This synergy between unstructured data processing and structured knowledge representation holds considerable promise for accelerating POM discovery and innovation (Figure 3).

### 5.2 Computer Vision and Interactive Data Visualization

Although computer vision may not be regarded as indispensable for advanced POM research, a substantial amount of published POM data is locked in graphical formats. Recently, large‐language models with vision capabilities (e.g., GPT‐4V) have demonstrated the ability to parse and extract experimental data from figures—including thermogravimetric analysis curves and redox states—in the context of MOFs.^[^ ^55^ ^]^ Adapting such vision models to the distinct challenges of POMs, where reaction progress may hinge on subtle color changes, precipitation onset, or intermediate formation, is a promising yet underexplored research direction.

Many modular platforms for POM synthesis rely on visual cues—such as turbidity or precipitate formation—to guide key steps like filtration. Recent advances from the Hein group demonstrate how webcam‐based systems, driven by deep learning, can monitor reactions in real time by detecting color changes linked to redox states or precipitation events.^[^ ^56^ ^]^ Automated platforms (e.g., HeinSight 2.0) can thus promptly identify turbidity or crystallization endpoints, allowing for timely reaction control. In POM chemistry, where precise control over reaction variables is essential, and thus AI‐driven vision can be potentially integrated with other sensors (e.g., pH and redox potential) to adjust reagent feeds or stirring conditions automatically. This can reduce researcher workload and enhance reproducibility.

Interactive data visualizations are effective tools for analyzing high‐dimensional and time‐series data in chemistry. Incorporating human‐in‐the‐loop workflows supports exploration, insight generation, hypothesis testing, and result confirmation. Visual analytic systems,^[^ ^57^ ^]^ for instance, combine data visualization, machine learning, and user interaction to transform raw data into actionable information. Techniques such as visually comparing subspaces in large datasets^[^ ^58^ ^]^ or identifying correlations in chemical^[^ ^59^ ^]^ and biomolecular data^[^ ^60^ ^]^ can reveal patterns that automated algorithms may potentially miss. Visualization methods also help researchers interpret and guide machine learning models,^[^ ^61^, ^62^, ^63^ ^]^ and they are no longer confined to desktop environments, that augmented and virtual reality setups,^[^ ^64^ ^]^ for example, offer immersive ways to navigate complex molecular assemblies. Collectively, these visual analytics techniques and computer‐vision capabilities place humans at the center of data analysis, bridging the gap between large‐scale data pipelines and on‐the‐fly experimental decision‐making in POM research.

### 5.3 Data Infrastructure and Community Efforts

The adoption of FAIR (Findable, Accessible, Interoperable, Reusable) data principles is crucial for advancing POM chemistry.^[^ ^65^ ^]^ Currently, many computational results already conform to standardized formats,^[^ ^66^ ^]^ which facilitates FAIR data sharing. However, compared to other domains of materials science,^[^ ^47^ ^]^ POMs have yet to benefit from large‐scale quantum mechanical calculations that generate the extensive datasets needed for data‐driven discoveries.

Experimental POM data are typically shared through publications and repository platforms, some of which may not share programmatic access or FAIR data sharing policies, thus requiring intense manual involvement. The growing use of robotic synthetic platforms is likely to change this, as improvements in reproducibility will allow researchers to report both successful and unsuccessful outcomes, which is essential information for refining machine learning models. Through systematic and transparent reporting of experimental data, POM chemistry can be more effectively integrated into the broader chemical data ecosystem, fostering collaborative innovation and paving the way for data‐driven breakthroughs. Journals, funding agencies, and the broader research community all play a crucial role in supporting these efforts.

A practical next step would be a community database that follows the curated and ontology‐driven style of Chemical Entities of Biological Interest (ChEBI) rather than the broad crowd‐sourced model. Every POM entry would carry a persistent identifier plus linked records for synthesis conditions characterization data electronic state and literature provenance. The schema can extend Chemical Markup Language with terms from the Ontologies for Chemistry initiative^[^ ^67^ ^]^ so that records are machine‐actionable. Programmatic access through application programming interfaces and Resource Description Framework dumps will feed knowledge graphs and learning algorithms. Open resources such as the Materials Project or the Open Reaction Database have shown that FAIR repositories coupled with agreed ontologies accelerate discovery and power autonomous laboratories.^[^ ^66^, ^68^ ^]^ A similar framework would allow POM researchers to capture both successful and null results and turn them into reliable training data for future models (Figure 3).

## 6 Outlook

Establishing a robust, data‐driven ecosystem for POM research will require both refined cheminformatics and flexible, automated experimental workflows. Open‐access databases that harmonize experimental and computational insights, complete with consistent descriptors for archetypes, protonation states, and electronic configurations, would enable algorithmic exploration of the POM landscape at an unprecedented scale. In parallel, reconfigurable robotic platforms can integrate *in situ* spectroscopic and scattering methods to capture the subtleties of POM formation, such as slow crystallization or complex redox processes. By feeding this continuous stream of real‐time data into advanced analytics, researchers can systematically map reaction parameters while minimizing the trial‐and‐error approach that often dominates POM synthesis.

**Figure 4:**
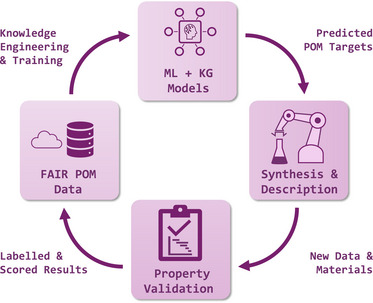
Closed loop workflow for data‐driven POM discovery. A FAIR POM database constructs and trains an ML and knowledge graph model which selects targets for robotic synthesis and *in situ* characterization. The labelled and uncertainty scored results return to the database and refine the next cycle.

Moreover, as summarized in Figure 4, community‐driven digital platforms can consolidate these outputs and incorporate AI tools for screening, predicting, and interpreting results, helping to narrow down the vast combinatorial space of potential POM architectures. This approach would also foster a collaborative research culture by encouraging the publication of both successful and partially explored reaction data, an important step toward FAIR‐compliant datasets. As computational power grows, closer integration of quantum chemical simulations within these pipelines will further improve predictions, allowing researchers to anticipate stable structural motifs or redox states before any reagents are mixed. Ultimately, by combining comprehensive data frameworks with responsive automation and targeted simulation, the field can move beyond serendipity and toward more, predictive POM design, opening new frontiers in catalysis, energy conversion, and (bio‐)materials innovation.

## Conflict of Interest

The authors declare no conflict of interest.

## Data Availability

Data sharing is not applicable to this article as no new data were created or analyzed in this study.
